# A tri-specific killer engager against mesothelin targets NK cells towards lung cancer

**DOI:** 10.3389/fimmu.2023.1060905

**Published:** 2023-02-22

**Authors:** Philippa R. Kennedy, Daniel A. Vallera, Brianna Ettestad, Caroline Hallstrom, Behiye Kodal, Deborah A. Todhunter, Laura Bendzick, Peter Hinderlie, Joshua T. Walker, Brittany Pulkrabek, Ira Pastan, Robert A. Kratzke, Naomi Fujioka, Jeffrey S. Miller, Martin Felices

**Affiliations:** ^1^Division of Hematology, Oncology, and Transplantation, Department of Medicine, University of Minnesota, Minneapolis, MN, United States; ^2^Department of Radiation Oncology, University of Minnesota, Minneapolis, MN, United States; ^3^Department of Obstetrics, Gynecology and Women’s Health, University of Minnesota, Minneapolis, MN, United States; ^4^National Cancer Institute, National Institutes of Health, Bethesda, MD, United States

**Keywords:** immunotherapy, lung, cancer, NK cell, TriKE, biologic, NSCLC

## Abstract

New treatments are required to enhance current therapies for lung cancer. Mesothelin is a surface protein overexpressed in non-small cell lung cancer (NSCLC) that shows promise as an immunotherapeutic target in phase I clinical trials. However, the immunosuppressive environment in NSCLC may limit efficacy of these therapies. We applied time-of-flight mass cytometry to examine the state of circulating mononuclear cells in fourteen patients undergoing treatment for unresectable lung cancer. Six patients had earlier stage NSCLC (I-IVA) and eight had highly advanced NSCLC (IVB). The advanced NSCLC patients relapsed with greater frequency than the earlier stage patients. Before treatment, patients with very advanced NSCLC had a greater proportion of CD14^-^ myeloid cells than patients with earlier NSCLC. These patients also had fewer circulating natural killer (NK) cells bearing an Fc receptor, CD16, which is crucial to antibody-dependent cellular cytotoxicity. We designed a high affinity tri-specific killer engager (TriKE^®^) to enhance NK cytotoxicity against mesothelin^+^ targets in this environment. The TriKE consisted of CD16 and mesothelin binding elements linked together by IL-15. TriKE enhanced proliferation of lung cancer patient NK cells *in vitro*. Lung cancer lines are refractory to NK cell killing, but the TriKE enhanced cytotoxicity and cytokine production by patient NK cells when challenged with tumor. Importantly, TriKE triggered NK cell responses from patients at all stages of disease and treatment, suggesting TriKE can enhance current therapies. These pre-clinical studies suggest mesothelin-targeted TriKE has the potential to overcome the immunosuppressive environment of NSCLC to treat disease.

## Introduction

In the United States, more than a million people were newly diagnosed with cancers of the lung and bronchus between 2015 and 2019 (1). This actually represents a decline in the incidence of the disease, in line with lower tobacco consumption. However, just under half of these cancers were diagnosed at a late stage with cells at distant sites, where five-year survival was as low as 7%. Outcomes for non-small cell lung cancer (NSCLC), which makes up more than 80% of lung cancers, have been improving with five year survival rates increasing from 16% in 1975 to 22% in 2012 (2). The last ten years have seen even more rapid increases in survival, coincident with improvements in early detection, targeted molecular therapy and immunotherapy (3–5). In particular, immune checkpoint inhibitors have demonstrated durable responses in patients with advanced disease, such as the Checkmate 017/057 trials where patients whose cancers had not progressed after 2 years had an 82% chance of surviving 3 years later (6). In these trials, 5-year survival rates were 13% for nivolumab, compared to 3% for chemotherapy alone. While this is an impressive response, it is still a minority of patients that benefit long term. Therefore, despite these improvements, more people die from lung cancer in the United States than from any other cancer (1). It remains a priority to design new interventions that can work alongside recent improvements in clinical practice.

Mesothelin is a 40 kDa surface glycoprotein that was identified in a screen for cancer antigens using the ovarian cancer line OVCAR3 (7, 8). These initial studies found mesothelin in ovarian cancers, mesothelioma and pancreatic cancers, but not lung cancer. Expression in healthy tissues was restricted to mesothelial cells lining the pleura, peritoneum and pericardium, suggesting it might be used to target these cancers with limited side effects. Later screening with an antibody clone that worked on fixed tissue sections (5B2) revealed mesothelin is absent from small cell lung cancer, but is expressed by approximately half of lung adenocarcinomas (9, 10). A higher affinity clone with an overlapping epitope (SS1) was subsequently developed for therapeutic use (11). In 2007, the suitability of SS1 for the treatment of lung cancer was demonstrated *in vitro* by immune responses generated against lung adenocarcinoma line, A549 (12), and an immunotoxin coupled version of this antibody has further been investigated in clinical trials (NCT01051934, NCT00066651, NCT00006981, NCT01362790).

While the function of mesothelin is incompletely understood, it is known to bind CA125 and promote cellular interactions. This may relate to why expression of mesothelin, as assessed by immunohistochemistry, is linked to worse prognosis in a number of different cancers, but particularly in lung adenocarcinoma (13, 14). Thus, the limited expression of mesothelin in non-essential tissues; high expression in cancers; and a presumed role in cancer progression; have made it an attractive target of cancer immunotherapies. A number of clinical trials have been initiated to investigate the safety and efficacy of targeting mesothelin (15). Strategies include monoclonal antibody therapies, immunotoxins, vaccines and chimeric antigen receptor (CAR) cellular therapies, all directed against the mesothelin antigen. There have been successes and setbacks. Initial concerns regarding the on-target off-tumor effects on healthy mesothelial cells, were unfounded in the case of pericarditis, but dose limiting in the case of pleuritis. Overall, there has been a failure to identify patients who will benefit from treatments, or optimal combinations of therapies. While mesothelin targeting still holds promise in the treatment of lung cancer, new approaches are necessary to enhance efficacy whilst limiting toxicity.

One of the challenges to successful immunotherapy is an impaired immune system in patients with cancer. Natural killer (NK) cells are innate lymphoid cells that spontaneously kill stressed or diseased cells, using a variety of germline-encoded receptors. In addition to their rapid cytotoxic responses, NK cells secrete cytokines to activate the adaptive immune system – making them an important therapeutic target in the treatment of cancer (16, 17). Several studies have noted that the number of NK cells in peripheral blood is decreased in NSCLC patients compared to healthy controls (18–20). In response to chemotherapy (18) and immunotherapy, numbers of circulating NK cells decrease, except in those patients that respond well to treatment (21). Indeed, high numbers of circulating NK cells prior to treatment is predictive of clinical response in advanced NSCLC patients (22, 23). These circulating cells are presumably indicative of the tumor microenvironment, where it has been shown that a high density of infiltrating NK cells is associated with better overall survival (24, 25). We hypothesized that impaired NK cell responses could be limiting mesothelin-targeted therapies. If NK cell responses can be boosted in addition to enhanced targeting of mesothelin^+^ lung cancer cells, this dual improvement in immune responses has the potential to succeed where other strategies targeting mesothelin have failed. Tri-specific killer engagers (TriKE^®^) are trivalent molecules that redirect killing of NK cells against a target antigen and increase NK cell functions through an IL-15 moiety (26, 27). We set out to investigate the state of the immune system in patients undergoing chemotherapy and immunotherapy for non-resectable lung cancer. Having identified potential impediments to therapy in those patients, we tested a novel anti-mesothelin TriKE and demonstrated its efficacy *in vitro*, using immune cells isolated from patient blood.

## Materials and methods

Detailed protocols are available as a collection at protocols.io (dx.doi.org/10.17504/protocols.io.5qpvorx29v4o/v1).

### Primary tissue

Blood from healthy donors were procured from the Memorial Blood Bank (Minneapolis, MN). Peripheral blood mononuclear cells (PBMC) and NK cells were isolated as described (dx.doi.org/10.17504/protocols.io.81wgbp94yvpk/v1). In brief, ficoll separation of mononuclear cells was performed on Leukopak samples and NK cells were isolated using negative-selection magnetic beads (EasySep Human NK Cell Enrichment Kit; STEMCELL Technologies) routinely giving 90-95% purity. Samples from patients undergoing treatment for metastatic or unresectable lung cancer were obtained from Masonic Cancer Center Thoracic Translational Working Group Lung Cancer and Pulmonary Nodule Biorepository. Viably frozen mononuclear cells were obtained at baseline, post treatment and upon progression. Patient characteristics are listed in **Tables 1** and **S1**. All samples were de-identified and their use was approved by the University of Minnesota and NMDP institutional review board in accordance with the Declaration of Helsinki (STUDY00002255).

**Table 1:** Patient characteristics.

	Cancer	NSCLC (Stage I-IVA)	NSCLC (Stage IVB)	Small cell
	Patients	6	8	3
	Median age (Interquartile range)	65 (62-70)	67 (55-69)	61 (57-67)
Sex	Female	3 (50%)	2 (25%)	1 (33%)
	Male	3 (50%)	6 (75%)	2 (67%)
Ethnicity	White	6 (100%)	7 (87.5%)	2 (67%)
	Mexican		1 (12.5%)	
	Asian			1 (33%)
Smoking	Never	0	2 (25%)	0
	Former	5 (83%)	5 (82.5%)	2 (67%)
	Current	1 (17%)	1 (12.5%)	1 (33%)
Staging	I-III	4 (67%)		
	IVA	2 (33%)		
	IVB		8 (100%)	
	Limited			1 (33%)
	Extensive			2 (67%)
Histology (differentiation status)	Adenocarcinoma	5 (2 poorly differentiated, 1 poor-moderate, 2 not specified)	7(3 poorly differentiated, 1 mucinous, 1 moderate, 1 well, 1 not specified)	
	Adeno-squamous	1 (poorly differentiated)		
	Squamous		1 (poorly differentiated)	
	Small cell			3
	PDL1 <50%	4/5	6/8	n/a
Treatments	Chemotherapy	6 (100%)	5 (63%)	3 (100%)
	Radiation	5 (83%)	6 (75%)	2 (67%)
	Checkpoint inhibitor*	6 (100%)	5 (63%)	1 (33%)
	Targeted therapy	1/6 (17%)	3/8 (38%)	0/3 (0%)
	Steroids**	1 (17%)	1 (13%)	0 (0%)
Complications	Immune-related adverse events	5/6 (83%)	3/3 (100%)	n/a
Progression	Progression (Y/N)	3/6 (50%)	7/7 (100%)	1/3 (33%)
	Died	0/5 (0%)	3/8 (38%)	1/3 (33%)
	Median time to 1st progression (months)	5.6 (5.4-7.1)	7.9 (3.5-16.9)	9.1 (n/a)
Type of progression	Systemic	3/3	6/7	0/1
	Central nervous system only	2/3	2/7	1/1
Best response to initial therapy	Complete response	2/6	0/7	0/3
	Partial response	5/6	5/7	3/3
	Stable disease	2/6	2/7	0/3
	Progressive disease	0/6	2/7	1/3
Lost to follow up <1 year		1/6	0/8***	0/3

Patients with small cell lung cancer are only included in supplementary data.

* checkpoint blockade includes pembrolizumab, durvalumab, nivolumab, atezolizumab and ipilimumab.

** at least 10mg prednisone at the time of a sample collection.

*** two died within a year, one within a few months precluding follow up.

### Cell lines

Details of culture for the following cells lines are provided at protocols.io (dx.doi.org/10.17504/protocols.io.rm7vz82o8vx1/v2). Lung adenocarcinomas: A549 (RRID : CVCL_0023, American Type Culture Collection, ATCC), NCI-H322 (RRID : CVCL_1556, Sigma Aldrich), NCI-H522 (RRID : CVCL_1567, UNKOWN); large cell lung carcinoma: NCI-H460 (RRID : CVCL_0459, ATCC); chronic myelogenous leukemia line, K562 (RRID : CVCL_0004); and the NK-like cell line NK-92 (RRID : CVCL_2142). All cell lines were routinely checked for mycoplasma infection.

### TriKE production

The design and production of second generation TriKE has been described previously (27). DNA shuffling and ligation techniques connected a camelid anti-CD16 complementarity determining region spliced into a humanized scaffold; *via* linker regions to human IL-15; and then on to anti-mesothelin scFv. The latter sequence, obtained from Ira Pastan (National Institute of Health, Maryland, USA), was originally derived from SS1 (28), but refined through affinity maturation (29) and stabilized with disulphide bridges (30). The resulting in-frame sequence was confirmed by the Biomedical Genomics Center, University of Minnesota, Minnesota, USA sequencing service. Protein was isolated and refolded from bacterial inclusion bodies. It was purified through ion exchange (Q Sepharose Fast Flow Columns) and size exclusion (Superdex 200, GE Healthcare). Purity was determined *via* SDS PAGE stained for protein (Simply Blue Safe Stain, Invitrogen).

### Mass cytometry time of flight

Mass cytometry time of flight (CyTOF) analysis of freshly defrosted patient PBMC was performed on a CyTOF 2 analyzer (Fluidigm), as described (dx.doi.org/10.17504/protocols.io.5qpvo522dl4o/v1). The antibodies used for CyTOF are listed in **Table S2**. For differential abundance analysis, within live (cisplatin -) single cell (IR) leukocytes (CD45+), NK cells are considered to be CD56+ CD3- CD19- CD33-; while myeloid cells are CD33+ CD3- CD19-.

### Flow cytometry-based assays

Cytokine production and degranulation – as measured by the appearance of LAMP1/CD107a at the cell surface and accumulation of intracellular cytokines, was performed on freshly isolated PBMC and analyzed on a flow cytometer (LSR II, BD Biosciences), gating on live CD56+ CD3- cells to identify NK cells, as described (dx.doi.org/10.17504/protocols.io.8epv51me6l1b/v1). Proliferation of NK cells in enriched populations or amongst PBMC were measured by the dilution of a permanent membrane dye by flow cytometry (dx.doi.org/10.17504/protocols.io.261geojwol47/v1).

### Bioavailability of IL-15

The bioavailability of the IL-15 component of the TriKE was assessed using a fluorescent redox-sensitive viability dye, resazurin (R&D Systems Cat. No. AR002), and NK-92, as measured on a fluorescence plate reader (Tecan; dx.doi.org/10.17504/protocols.io.yxmvmxqw9l3p/v1). Based on this measurement the ‘equifunctional’ dose of IL-15 for the TriKE is that which induces an equivalent amount of NK92 stimulation (i.e 0.67 nM IL-15 and 30 nM cam1615SS1).

### Live-imaging cytotoxicity

In order to track NK cell cytotoxicity, NCI-H460 and A549 were stably transfected with IncuCyte^®^ NucLight Red Lentivirus Reagent (Essen Bioscience Cat. No. 4625). Time-lapse imaging of these cells was performed in the presence of enriched NK cells and an indicator of apoptosis (Caspase 3/7 Green Apoptosis Assay Reagent, Cat. No. 4440, Essen Bioscience) at 30 min intervals in an IncuCyte^®^ S3 (Essen Biosciences), as described (dx.doi.org/10.17504/protocols.io.q26g7b6x8lwz/v1). The percentage of live targets at each time point was normalized to the growth of those targets alone. The equifunctional dose of IL-15 used in this assay was calculated using the bioavailability of IL-15 assay listed above.

### Chromium release cytotoxicity

For greater sensitivity of cytotoxicity by thawed patient PBMC that contain a small population of NK cells, we measured chromium (Cr^51^) release from NCI-H460 cells. PBMC were thawed and rested overnight in RPMI media with 10% fetal bovine serum and 100 U/mL penicillin and streptomycin (all Gibco). The following day, NCI-H460 cells were labelled with 200 mCi sodium chromate-Cr^51^ (DuPont, Wilmington, DE) for 60 minutes. Effector-to-target ratios were setup at 20:1, 6.7:1 and 2.2:1 with the indicated drugs. All determinations were done in triplicate, and specific lysis after 4 h was determined as a percentage ((experimental mean cpm – spontaneous release mean cpm)/(total release mean cpm – spontaneous release cpm) x 100%), where cpm refers to gamma counts per minute.

### Synaptic polarization by microscopy

Confocal microscopy was performed using a Nikon A1R microscope, using 60x 1.27 NA objective with 488 nm and 637 nm laser excitation, detected using GaAsP detectors and a transmitted light detector. A549 were labelled with CellTrace Far Red (Thermo Fischer Scientific) and allowed to adhere to chamber slides (Ibidi) overnight at 37°C 5% CO_2_. NK cells and the indicated drugs were added to wells containing A549 and incubated for 5 min at 37°C 5% CO_2_, before being fixed with 4% paraformaldehyde/PBS (Electron Microscope Solutions) for 30 min at room temperature. Cells were permeabilized in 0.01% Triton X/PBS for 5 min at room temperature. Cells were washed with PBS, then incubated for 1 h at room temperature in blocking solution (3% bovine serum albumin, 1% human AB serum/PBS). Primary antibodies against pericentrin (Abcam Cat# ab4448, RRID : AB_304461) and AlexaFluor488-conjugated antibody against perforin (BioLegend Cat# 308108, RRID : AB_493252) were incubated in blocking solution for 1 h at room temperature. Cells were washed again in PBS, then were incubated with a blocking solution containing AlexaFluor647plus-conjugated anti-rabbit antibody (1 µg/ml, Thermo Fisher Scientific Cat# A32849, RRID : AB_2762840) for 1 h at room temperature. Cells were washed and post-fixed with 4% paraformaldehyde/PBS for 5 min at room temperature and stored at 4°C prior to imaging. Conjugates were identified in the transmitted light channel and a z-stack through the contact point was acquired. Images were analyzed using ImageJ (RRID : SCR_003070). The microtubule organizing center (MTOC) was identified as the AlexaFluor647plus signal within the NK cell. Perforin granules were qualitatively scored 1 (majority of granules are gathered at the MTOC) or 0 (granules are dispersed throughout the cell). The shortest distance between the MTOC and the synapse was measured and normalized to the distance between the synapse and the back of the cell for the z-slice where the MTOC appeared.

### *In vivo* xenogeneic adoptive transfer model

NSG mice (NOD.Cg-Prkdcscid Il2rgtm1Wjl/SzJ, RRID:IMSR_JAX:005557; n=6/group; 3 females, 3 males) underwent whole body X-ray irradiation with 225 cGy to facilitate human cell engraftment. One day later they were injected intravenously with 750,000 GFP/luciferase-expressing NCI-H460 cells. Bioluminescent imaging was performed two days after tumor engraftment to balance the mice evenly into three groups (‘day 0’). Freshly isolated PBMC were depleted of CD3+ and CD19+ cells using magnetic beads to obtain a population enriched for NK cells and then cultured overnight with 10 ng/mL IL-15. The NK cell proportion (CD56+ CD3-) within the cell population was quantified by flow cytometry after overnight culture and the dosage was balanced so that, three days after tumor engraftment (‘day 1’), each mouse received 1 million intravenous NK cells. A control group of mice received no NK cells (H460 alone). Mice receiving NK cells were dosed with cam1615SS1 (25 µg/dose daily, five days a week for two weeks) or an equifunctional dose of IL-15 (133.8 ng/dose daily, five days a week for two weeks), delivered by the intraperitoneal route. Bioluminescent images were taken at day 0 and day 7 and mouse blood was drawn on day 13 and day 20. These blood samples were stained for human NK cells (murine CD45-, human CD45+, CD56+, CD3-, CD16, Ki67, CD69) to monitor their activity.

### Software and statistics

Power and sample size calculations of lung cancer patients analyzed by CyTOF were performed with PS software version 3 (31). ViSNE analysis was performed in Cytobank (Cytobank Inc., RRID: SCR_014043). Flow cytometry analysis and CyTOF sample concatenation were performed in FlowJo (Tree Star, Inc., RRID : SCR_008520). Differential abundance analysis of immune cell subsets within CyTOF samples was performed by Astrolabe Diagnostics. Microscopy analysis was performed in ImageJ (RRID : SCR_003070). All other statistical analyses were performed in GraphPad Prism (RRID: SCR_002798, version 8).

## Results

### Myeloid cells and NK cells are distinct in patients with advanced NSCLC

**Figure 1:**
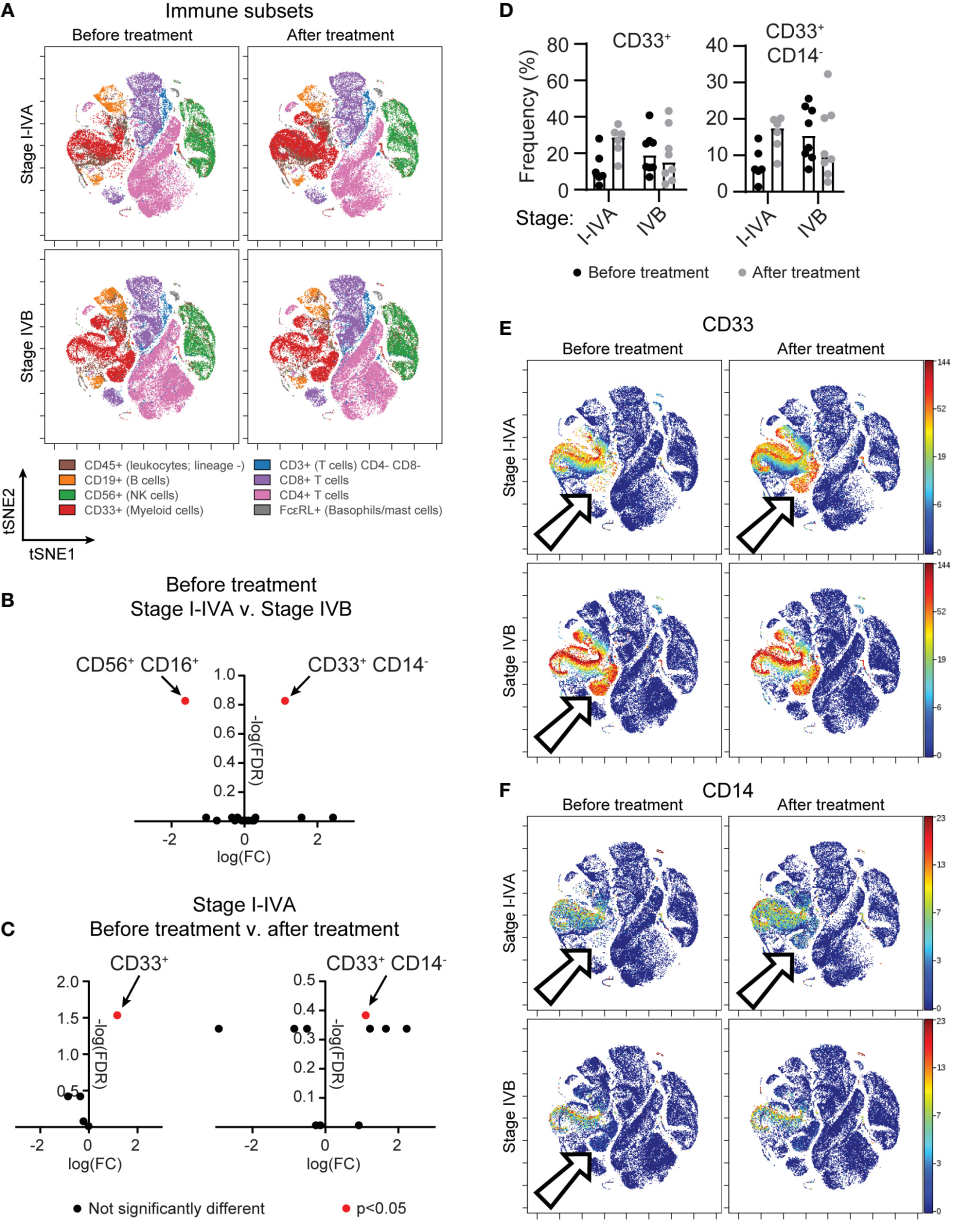
A population of circulating CD33+ CD14- myeloid cells is found in advanced NSCLC patients and after treatment onset. PBMC from donors with unresectable lung cancer were obtained from the thoracic/lung nodule biobank. Patients were grouped according to a diagnosis (see **Table 1**) of stage I-IVA (n=6) or stage IVB (n=8) NSCLC. Matched samples were obtained for all patients before they began therapy and after initial treatment began. **(A)** tSNE plots show concatenated NSCLC patient samples before or after treatment onset, analyzed by CyTOF, for all CD45+ live cells, highlighting immune subsets as indicated by the cell surface markers. **(B)** A differential abundance analysis of immune cell subsets (Astrolabe Diagnostics) was performed comparing I-IVA and IVB stage NSCLC patient samples before treatment onset. The log fold change (x axis) and negative log false discovery rate (y axis) for immune subsets is plotted. Positive fold changes indicate a population is larger in stage IVB NSCLC. **(C)** A differential abundance analysis of stage I-IVA NSCLC patients before or after treatment. The graph on the left shows the analysis when considering only 5 immune subsets (T cells, B cells, NK cells, myeloid cells or others), the graph on the right shows the same analysis when considering smaller subsets within these gross definitions. Positive fold changes indicate a population is larger after treatment onset. In both **(B, C)** significantly altered immune cell subsets (p<0.05) are highlighted in red. **(D)** The frequency of CD33+ cells and CD33+ CD14- cells as a proportion of all CD45+ cells is plotted for all patient groups across both timepoints. **(E, F)** tSNEs shown in **(A)** are colored according to the intensity of **(E)** CD33 or **(F)** CD14 staining. Arrows point to a CD33+ CD14- ‘island’ that is absent in stage I-IVA NSCLC patients before treatment, but present upon treatment; and is present in stage IVB NSCLC patients.

Peripheral blood mononuclear cells (PBMC) of patients undergoing treatment for non-resectable NSCLC were obtained from the Lung Cancer and Pulmonary Nodule Biorepository (University of Minnesota). Samples were collected prior to initial treatment, after initial treatment (chemotherapy, radiation, immune checkpoint inhibitors) and at relapse, if applicable. Patient characteristics are summarized in **Table 1**. To understand the state of the immune system in patients undergoing current standard-of-care treatments, we applied time-of-flight mass cytometry (CyTOF) for in depth characterization of immune subsets (**Figure 1A**), with a particular focus on the NK cell phenotype. The panel comprised established markers of immune subsets: T cells (CD3, CD8, CD4, FoxP3, CD25), B cells (CD19), NK cells (CD56), monocytes (CD33, CD14) and mast cells/basophils (FcϵRI); in addition to 23 markers of maturation, activation and proliferation (**Table S2**). A comparison of stage I-IVA and stage IVB NSCLC patients revealed differences in myeloid cells and NK cells prior to treatment onset (**Figure 1B**). Highly advanced NSCLC patients had more CD33+ CD14- myeloid cells than patients at an earlier disease stage (differential abundance analysis, p=0.013). However, there were increases in myeloid populations in stage I-IVA NSCLC patients after treatment onset, with an increase in the CD33+ myeloid population overall (p=0.0058; **Figure 1C**), particularly the CD33+ CD14- population (p=0.024; **Figures 1C–F**). These CD33+ CD14-low CD11b-low myeloid cells have features of early stage myeloid-derived suppressor cells (32). Increased levels of circulating MDSC are associated with lower overall survival in a number of different cancers (33).

**Figure 2:**
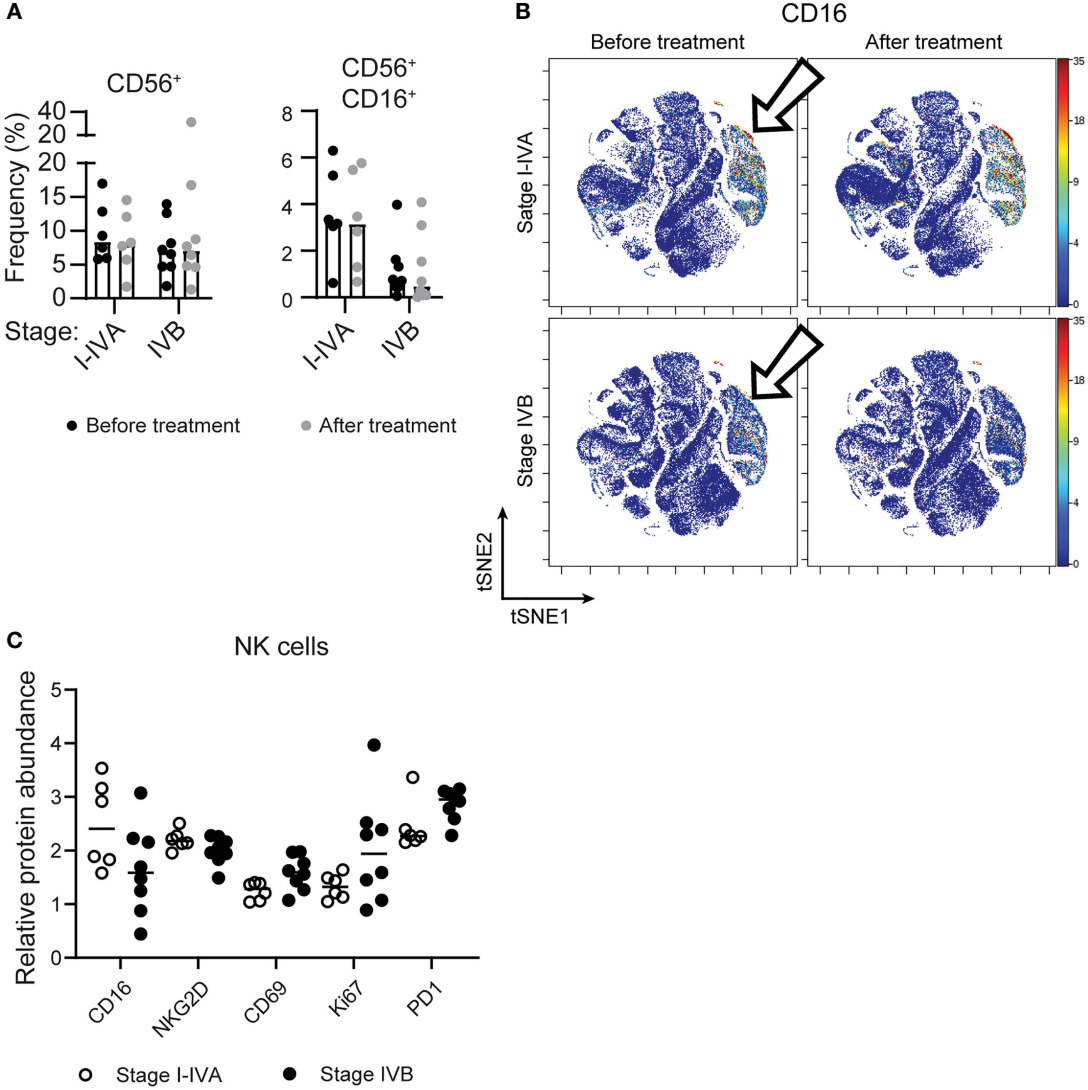
Later stage NSCLC patients have lower expression of CD16 on circulating NK cells. **(A)** The frequency of CD56+ NK cells and CD56+ CD16+ NK cells as a proportion of all CD45+ cells is plotted across patient groups. **(B)** tSNEs of concatenated NSCLC patient data, as in **Figure 1**, are colored according to CD16 expression, which is significantly different on the NK cell subset (arrows). **(C)** The relative protein abundance (Astrolabe diagnostics) is plotted on all CD56+ cells from NSCLC patients before treatment for CD16, NKG2D, CD69, Ki67 and PD1.

Examining the circulating NK cells in patients at different stages of disease revealed similar numbers of CD56+ NK cells at all stages (**Figure 2A**), however there was lower expression of CD16/FcγRIII on NK cells from later stage NSCLC patients, with more CD56+ CD16+ cells found amongst stage I-IVA NSCLC patients (p=0.018; **Figure 1B**, **2A–C**).

Included in this lung cancer dataset were three patients with small cell lung cancer (**Figure S1**). Since mesothelin has not been described on small cell lung cancer (SCLC), these patients were not as relevant to our examination of TriKE function. However, it was interesting to note that the CD33+ CD14- population was observed in these patients and the proportion of NK cells amongst circulating leukocytes decreased after treatment. Given the small size of this group, this was not statistically significant for all CD56+ cells or for CD56+ CD16+ cells, but was different for CD56+ CD16- cells which make up the majority of the CD56+ group in this case (differential abundance analysis, p=0.031). Altogether, there were very few NK cells of any sort circulating in SCLC patients after treatment onset.

Together these data suggest that the immune environment in which current therapies are applied to lung cancer patients is potentially suppressive, with high frequencies of myeloid-derived suppressive cells (MDSC) at advanced stages of disease and after treatment onset. In addition, the reduced expression of CD16 on NK cells for the most at risk NSCLC patients (stage IVB) has the potential to impact antibody-based therapies that utilize this Fc receptor to mediate a cytotoxic effect.

### TriKE induces NK cell proliferation *in vitro*

We designed a TriKE molecule targeting mesothelin to overcome the immunosuppressive tumor environment implicated by circulating immune cells in NSCLC patients. The molecule consists of three elements: a mesothelin-binding single chain variable fragment (scFv) based upon the therapeutic antibody SS1 (28), recombinant IL-15 moiety and a humanized single domain antibody (sdAb) against CD16; all connected by short linker sequences (34). This second generation TriKE encodes wild-type recombinant IL-15 for enhanced NK cell survival and proliferation, and a humanized camelid sdAb binds CD16 with greater stability than our first generation scFv fragment (27). Initial evaluation of the capacity of the TriKE to induce proliferation of an IL-2/IL-15 dependent cell line, NK92, revealed at equimolar quantities the TriKE was 45-fold less potent than recombinant human IL-15, presumably because of tertiary structure issues generated during the purification process (**Figure S2**).

**Figure 3:**
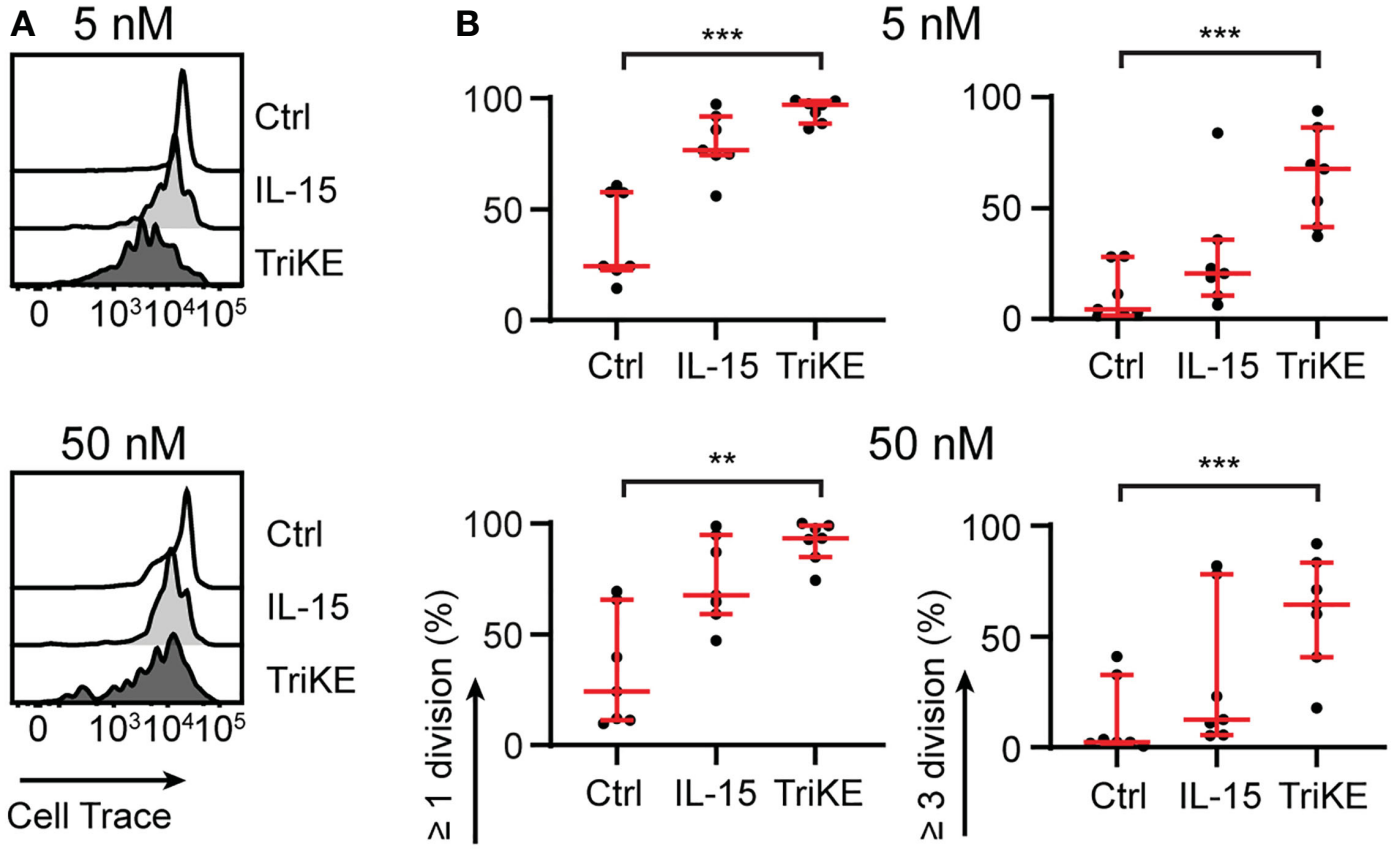
Cam1615SS1 promotes NK cell proliferation. **(A, B)** PBMC or purified NK cells were labelled with CellTrace Violet and cultured in media without drugs (Ctrl), with IL-15 or with cam1615SS1 (TriKE) at 5nM (top row) or 50nM (bottom row). After 7 days, the proliferation (CellTrace dye dilution) of NK cells (CD56+ CD3-) was assessed by flow cytometry. Representative histograms of CellTrace dye for one donor **(A)** are shown alongside quantification of cell division for multiple donors **(B)**. Ctrl, IL-15 and TriKE (n=7) analyzed by Friedman test with Dunn’s multiple comparisons. ** p<0.01, *** p<0.001.

We tested the ability of the TriKE to induce proliferation of NK cells from healthy human donors (**Figure 3**). PBMC were labeled with CellTrace Violet and the dilution of this dye was measured by flow cytometry for the CD56+ CD3- population of NK cells after 7 days. The TriKE produced comparable levels of proliferation to IL-15 at both 5 nM and 50 nM concentrations, which was significantly higher than unstimulated controls. At the end of the 7 days the cells were also assessed for viability as indicated by a live/dead membrane dye and annexin V staining (**Figure S3**). The 5 nM concentration of TriKE had no effect on viability compared to IL-15 or unstimulated controls, but the 50 nM concentration of TriKE showed lower viability and yield of NK cells than unstimulated controls, perhaps as a result of activation-induced cell death.

### TriKE enhances NK cell mTOC polarization towards lung cancer cells

**Figure 4:**
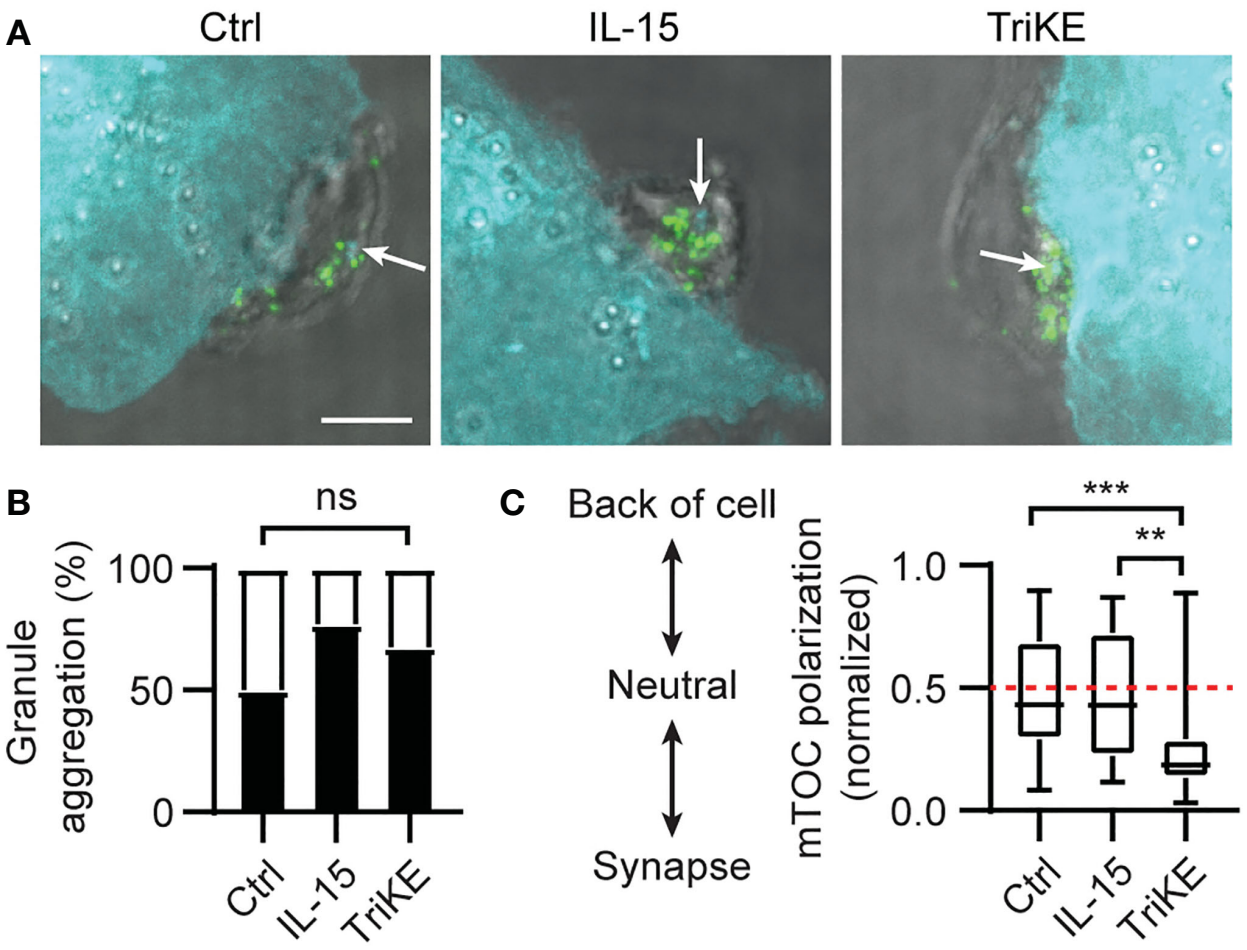
Cam1615SS1 promotes mTOC polarization. **(A–C)** NK cells (unlabelled) were incubated with the indicated drugs and A549 (CellTrace Far Red; cyan) for 5 mins, before being fixed and stained for the microtubule organizing center (mTOC; cyan in NK cells and white arrows) and perforin (green). Representative maximum intensity projections through each cell are shown **(A)** with scale bar 5μm (3 independent donors, at least 27 cells/condition). **(B)** Quantification of granule aggregation around the mTOC across all images; each cell was given a binary score for aggregated (1) or dispersed (0). Fisher Exact Test. **(C)** Quantification of the distance of the mTOC to the synapse as a ratio of the cell width. Kruskal-Wallis test with Dunn’s multiple comparisons. ** p<0.01, *** p<0.001.

Having confirmed the impact of the TriKE molecule on NK cell expansion, we went on to test its effect on cytotoxicity and cytokine production by NK cells in healthy human donors. The cytotoxic process in NK cells is separated into discrete steps controlled by activating signals (35). Once the NK cell has recognized the tumor cell it gathers perforin- and granzyme-containing cytolytic granules around the microtubule organizing center (MTOC), before polarizing this structure to the contact point with the tumor cell (known as the synapse). From here, granules are released onto the tumor cell where the perforin forms pores in the tumor membrane. These pores allow granzymes to enter and induce caspase-dependent cell death. When we compared the synapses formed between NK cells from healthy donors and A549 (a lung adenocarcinoma line) fixed after 5 min of interaction (**Figure 4A**), the perforin granules had gathered around the MTOC to a similar extent in all experimental treatments (**Figure 4B**), but the MTOC polarized to the synapse more frequently in the case of TriKE treatment, compared to NK cells treated with IL-15 or left untreated (**Figure 4C**). This suggests that TriKE rapidly initiates the cytotoxic machinery compared to IL-15 treatment.

### TriKE enhances degranulation and cytokine production against lung cancer cells

**Figure 5:**
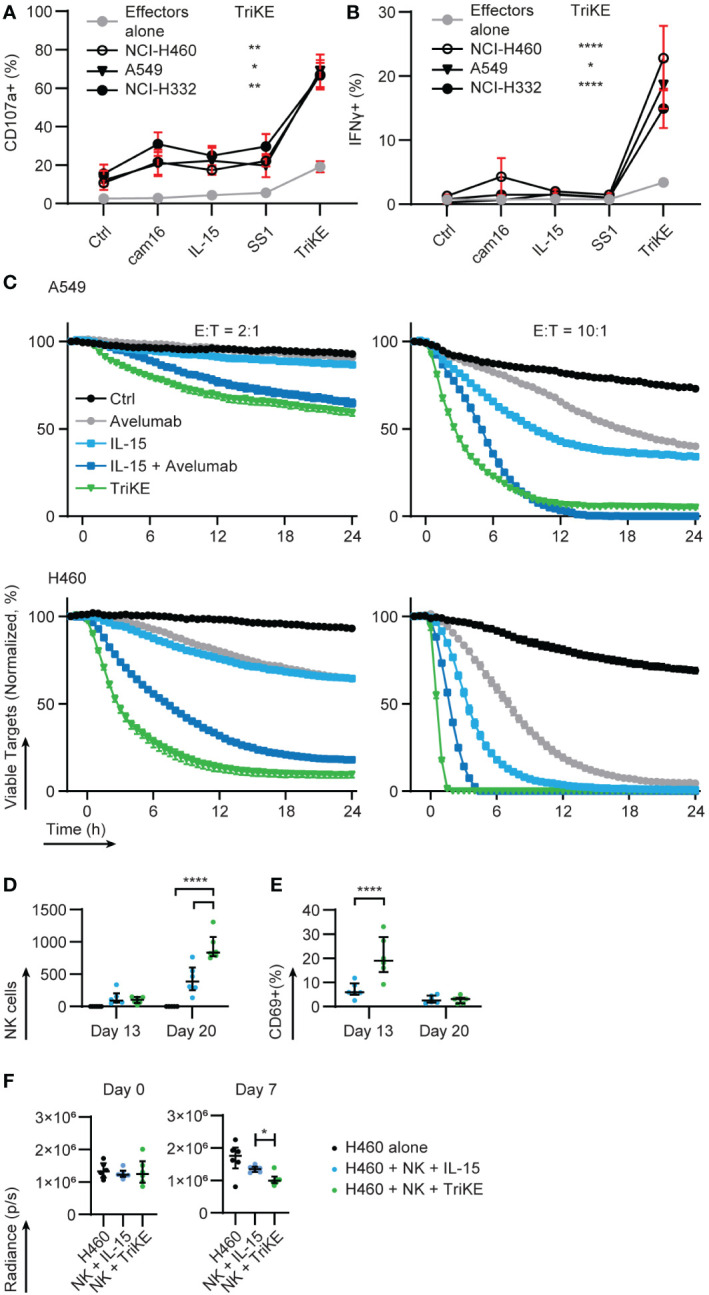
Cam1615SS1 enhances cytokine production, cytolysis and *in vivo* activation of NK cells. **(A)** PBMC were cultured in the presence of the indicated drugs (30nM) for 5 hours alone or in contact with lung cancer lines: NCI-H322, NCI-H460, A549. The amount of **(A)** degranulation (CD107a+) and **(B)** cytokine secretion (IFNγ) by NK cells (CD56+ CD3-) was assessed by flow cytometry. Effectors alone (Ctrl, IL-15, TriKE n=20; cam16 n=17; SS1 n=13), NCI-H322 (Ctrl, IL-15, TriKE n=14; cam16 n=11; SS1 n=10), NCI-H460 (Ctrl, IL-15, TriKE n=12; cam16 n=9; SS1 n=8), A549 (Ctrl, IL-15, TriKE, cam16, SS1 n=8). Bars show median and interquartile range. Analyzed by Kruskal-Wallis test with Dunn’s multiple comparisons. Significant differences are indicated by * p<0.05, ** p<0.01, **** p<0.0001 when comparing the effector alone with TriKE to the respective tumor condition with TriKE. **(C)** 10,000 fluorescent (NuclightRed) A549 (top row) or NCI-H460 (bottom row) were plated in triplicate with a fluorescent indicator of apoptosis (caspase 3/7 green) and allowed to adhere overnight. At time zero, purified NK cells were added to the wells at the specified effector to target cell ratios (E:T) with the indicated drugs (30nM TriKE; 0.67nM ‘equifunctional’ IL-15; 10μg/mL Avelumab). Cancer cell survival, normalized to the growth of cancer cells in wells without NK cells, was calculated on the basis of fluorescent cells that were not caspase 3/7+. One representative donor of at least three is shown. Bars show the mean and standard error of the mean for triplicates. **(D–F)** A metastatic xenogeneic model of lung cancer was established in NSG mice by intravenous injection of GFP/luciferase-expressing NCI-H460 cells. Three days after engraftment (‘day 1’), control mice were left untouched (black symbols), but test groups received 1 million human NK cells injected intravenously and treated with IL-15 treatment (blue symbols) or TriKE (green symbols). **(D)** The number of NK cells in blood samples drawn at two different timepoints was quantified by flow cytometry (mouse CD45-, human CD45+, human CD56+, human CD3-). **(E)** The proportion of these human NK cells with detectable surface CD69 was quantified by flow cytometry. **(F)** Tumor growth two days after engraftment (‘day 0’) and 6 days after NK cell dosing (‘day 7’) were quantified by bioluminescent imaging. Each dot represents an animal. Bars show the median and interquartile range. NK cell numbers and CD69 proportions were assessed by two-way ANOVA with Sidak’s multiple comparison test. Bioluminescent values for IL-15 and TriKE treatment were compared at day 0 and day 7 with a Mann-Whitney test. * p<0.05, ****p<0.0001.

NCI-H460 (large cell lung cancer), A549, NCI-H322 and NCI-H522 (adenocarcinomas) are NSCLC cell lines that express variable levels of mesothelin (12, 36). We challenged PBMC with these lines with or without TriKE or the individual elements that make up the TriKE (CD16 sdAb, IL-15, SS1 scFv) as controls. A flow cytometry-based assay was used to measure the accumulation of cytotoxic granule membrane protein, LAMP1/CD107a, at the surface of NK cells (as a proxy for granule release); while simultaneously detecting production of interferon γ (IFNγ) on a per NK cell basis after PBMC were co-incubated with these NSCLC lines for 5 h (**Figures 5A, B**, **S4**). The dose of TriKE was titrated using NCI-H460 and 30 nM was selected as an appropriate concentration for these *in vitro* assays (**Figure S5**). At this concentration, the TriKE was able to induce slight degranulation of NK cells amongst PBMC in the absence of tumor (median 19%) but this was dramatically increased when NK cells were challenged with NSCLC lines in the presence of TriKE (A549 52%, NCI-H460 81%, NCI-H322 78%, NCI-H522 61%). In comparison, these lines induced no significant degranulation of NK cells in the absence of TriKE (2%). Production of IFNγ was enhanced in NK cells against all three cell lines when challenged in the presence of TriKE (NK alone 3%, A549 14%, NCI-H460 13%, NCI-H322 10%, NCI-H522 21%). Together these data suggest TriKE can enhance anti-tumor activities of healthy NK cells against a range of NSCLC subtypes expressing mesothelin.

### TriKE enhances cytotoxicity of lung cancer cells

Time lapse imaging of NK cells and NSCLC lines was used to confirm that enhanced degranulation led to direct killing of mesothelin+ cells (**Figure 5C**). We compared the activity of the TriKE to IL-15, but also to a therapeutic checkpoint antibody, with or without co-treatment with IL-15, to determine if TriKE had greater therapeutic potential than this combination. Nivolumab and Pembrolizumab (anti-PD-1 human IgG4) are standard of care checkpoint inhibitors used in the treatment of NSCLC. However, given the mechanism of action of the TriKE, a better comparison would be Avelumab (anti-PD-L1 human IgG1), a checkpoint inhibitor that in addition to its checkpoint function also induces antibody-dependent cellular cytotoxicity by triggering CD16 on NK cells. We aimed to determine whether the co-delivery of IL-15 and CD16 signals from the TriKE, with its higher affinity interactions with CD16, would outperform individual signals from IL-15 and Avelumab. NCI-H460 and A549 were transduced with nuclear fluorescence to track their growth and a caspase 3/7 indicator revealed apoptosis in 24 h cultures, imaged every 30 min. When compared to the normalized growth of tumor alone, the addition of NK cells at two different effector to target (E:T) ratios had very limited effect on tumor growth (black lines). The addition of IL-15 (30 nM) or Avelumab (10 µg/ml) were each capable of reducing tumor growth (light blue and grey lines, respectively) with greater control when these therapies were combined (dark blue lines). However, the TriKE (30 nM) alone outperformed this therapeutic combination, rapidly eliminating NCI-H460 at the highest E:T ratios and reducing growth of the more resistant A549, even at lower E:T ratios (green lines).

### TriKE enhances NK cell activation *in vivo*

To test whether TriKE could induce human NK cell activation *in vivo*, we setup a xenograft mouse model of human lung cancer. NCI-H460-GFP/luciferase cells were injected intravenously into NSG mice and allowed to establish for three days and then were either left untreated (tumor alone control) or given a single intravenous dose of 1 million human NK cells. NK cell bearing mice were treated with TriKE or an equifunctional dose of IL-15 five days a week for two weeks. Blood was extracted from the mice on day 13 and day 20 and the presence of circulating human NK cells was analyzed by flow cytometry. There was a robust increase on day 20 in the relative number of human NK cells in animals treated with TriKE compared to those treated with IL-15 (**Figure 5D**). On day 13, the proportion of circulating human NK cells with detectable CD69 on their surface, which is an indicator of NK cell activation, was greater in TriKE-treated animals compared to IL-15-treated animals (**Figure 5E**). This model was also able to demonstrate a decrease in tumor load at day 7 in animals treated with TriKE compared to those treated with IL-15 (**Figure 5F**). Overall these data demonstrate the TriKE is able to activate healthy human NK cells *in vivo*.

### Healthy donor and lung cancer patient NK cells respond to TriKE

**Figure 6:**
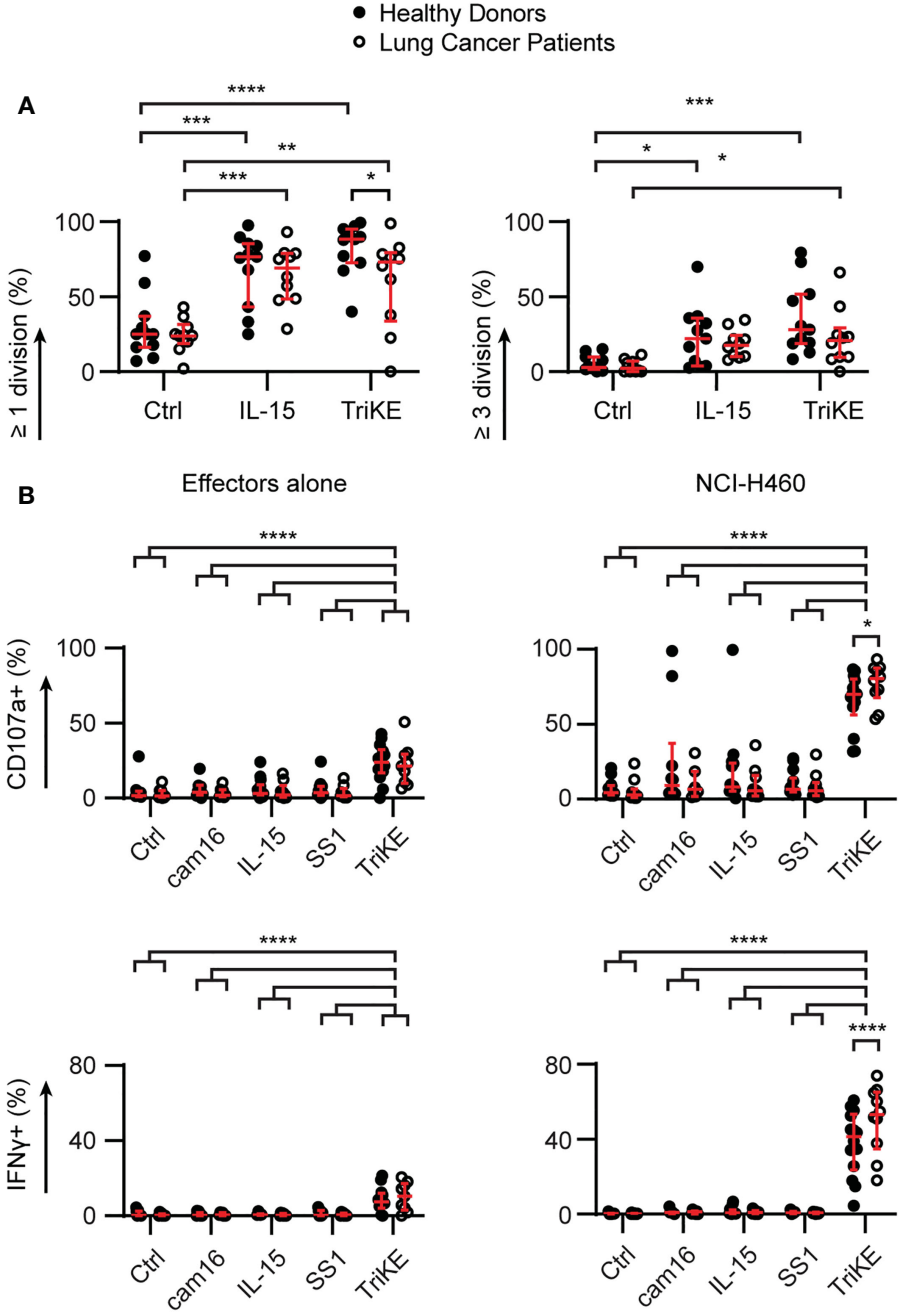
Cam1615SS1 promotes proliferation, cytokine production and degranulation of NK cells from both healthy donors and lung cancer patients. **(A)** PBMC were labelled with CellTrace Violet and cultured in media without drugs (Ctrl), with IL-15 or cam1615SS1 (TriKE) at 5nM. After 7 days, the proliferation (CellTrace dye dilution) of NK cells (CD56+ CD3-) was assessed by flow cytometry. Healthy donors (n=11) and lung cancer patients (n=10) were analyzed by Mixed effects analysis with Sidak’s multiple comparisons. **(B)** PBMC were cultured in the presence of the indicated drugs for 5 hours alone or in contact with lung cancer line NCI-H460. The amount of degranulation (CD107a+) and cytokine secretion (IFNγ) by NK cells (CD56+ CD3-) was assessed by flow cytometry; analyzed by mixed effects analysis with Sidak’s multiple comparisons. Healthy donors (cam16 n=10, all other conditions n=14); Lung cancer patients (cam16 n=7, all other conditions n=10). In all graphs, bars show median and interquartile range. * p<0.05, **p<0.01, *** p<0.001, **** p<0.0001.

Next, we examined whether NK cells from the peripheral blood of lung cancer patients would be capable of responding to TriKE similar to the response seen in healthy controls, given the immunosuppressive phenotype of circulating PBMC. PBMC were isolated from patients with NSCLC (described in **Table S1**) and challenged with TriKE alone, to examine the impact on proliferation and viability (**Figures 6A**, **S6A, B**), or in the presence of lung cancer lines, to examine the effect on degranulation and cytokine production (**Figure 6B**). The capacity of NK cells to undergo at least one division was slightly impaired for lung cancer patients compared to healthy controls. However, there was a significant increase in all measures of NK cell proliferation for lung cancer patients treated with TriKE compared to unstimulated controls, suggesting that NK cells in these patients can still respond to IL-15 stimulation. Surprisingly, these patients released more cytotoxic granules and produced more IFNγ when challenged with NCI-H460 in the presence of TriKE, compared to healthy controls. There was no increase in CD16 or other activating receptors in lung cancer patients compared to healthy controls that would explain this observation (**Figures S6C–F**). Together these data demonstrate that TriKE can enhance functional responses of NK cells in lung cancer patients.

### NK cells from patients with advanced disease still respond to TriKE

**Figure 7:**
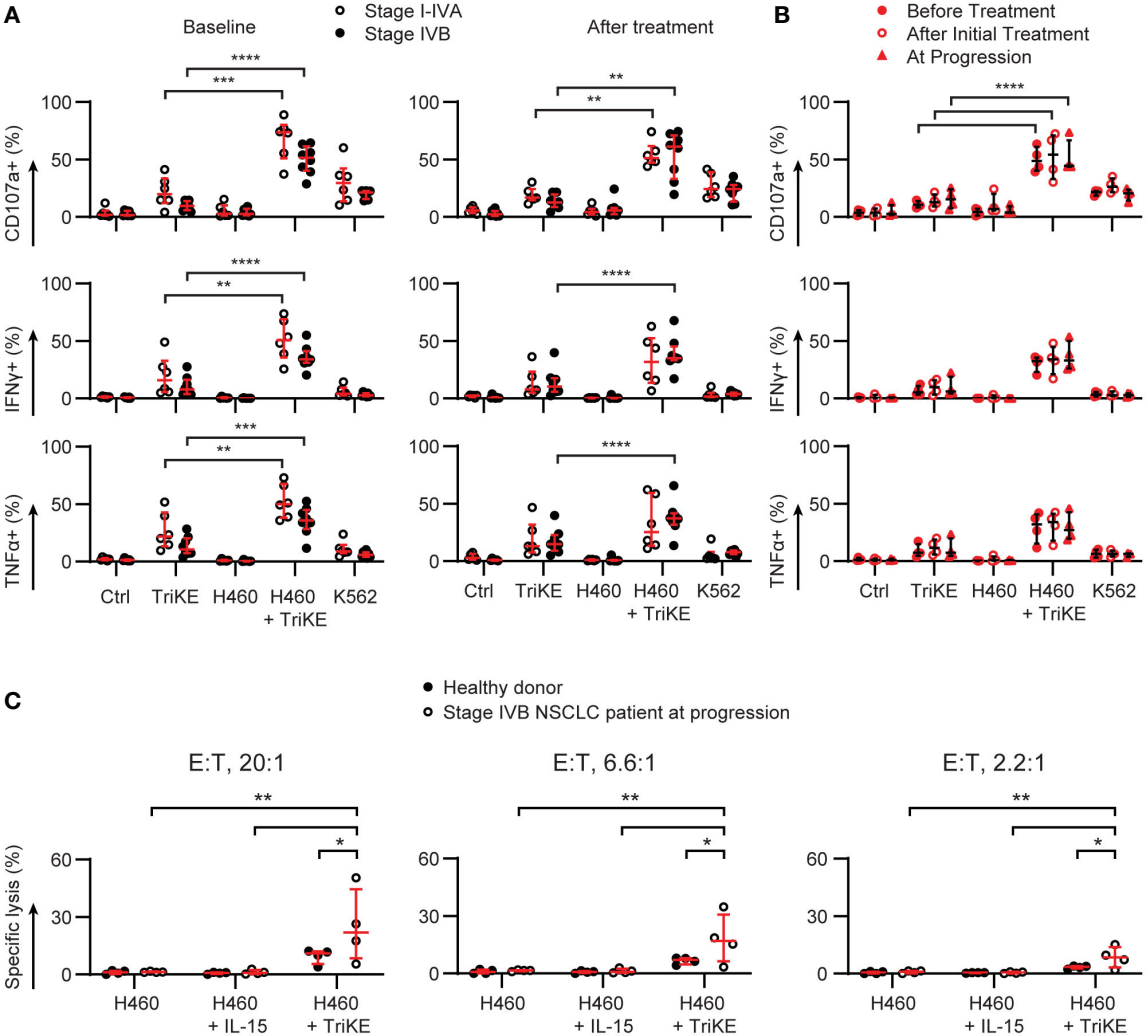
TriKE triggers responses against lung cancer cells by lung cancer patient NK cells at all stages of disease and treatment. PBMC from donors with non-resectable NSCLC were cultured for 5 hours alone (Ctrl), with a lung cancer cell line (NCI-H460) or a chronic myeloid leukemia cell line (K562) in the presence or absence of cam1615SS1 (TriKE). The amount of degranulation (CD107a+) and cytokine secretion (IFNγ and TNFα) by NK cells (CD56+ CD3-) was assessed by flow cytometry. **(A)** The data are organized according to the stage of treatment (before treatment – left; after treatment - right) or the disease stage (symbols, I-IVA n=6, IVB n=8). **(B)** The NK cell responses of four stage IVB NSCLC patients for whom progression samples were available are plotted according to the timepoint of treatment. **(C)** Lysis of NCI-H460 by PBMC was assessed in a 5 h chromium release assay with or without IL-15 or TriKE (both 30nM). Three effector to target (E:T) ratios were probed for healthy donors or the most at risk NSCLC patients (n=4). In all graphs, bars show the median and interquartile range. Analyzed by two-way repeated measures ANOVA with multiple comparison tests (highlighting just the TriKE and TriKE + H460 comparisons in A&B) * p<0.05, **p<0.01, *** p<0.001, **** p<0.0001.

We were particularly interested in NK cell responses to TriKE in patients with late-stage disease who went on to relapse at higher frequencies than patients with earlier stage NSCLC (**Table 1**), as this might be the target population for a clinical trial. PBMC from the patients examined in **Figures 1**, **2** were challenged with NCI-H460 to determine whether the immunosuppressive phenotype of circulating PBMC in these patients could be overcome (**Figures 7A, B**). The disease stage had a significant effect on the degranulation and TNFα production of the NK cells, at baseline, but not during treatment for NSCLC (two-way ANOVA, p<0.05). All patients had insignificant responses to NCI-H460 directly and had limited responses to a classic indicator of NK cell natural cytotoxicity, K562 (37). This chronic myeloid leukemia line lacks MHC class I that normally inhibit NK cell responses and is recognized by a number of germline encoded activating receptors normally expressed by NK cells. IFNγ and TNFα production triggered in NK cells against these tumor cells was significantly lower than that induced by TriKE in response to NCI-H460, for all disease stages (I-IVB) and timepoints (before treatment, after treatment or at progression). Degranulation was also significantly higher for the TriKE-driven responses to NCI-H460 than for any of the control conditions at all disease stages and timepoints. The maintenance of these responses, despite lower CD16 expression on NK cells from stage IVB NSCLC patients (**Figure 2**), suggests that the high affinity anti-CD16 sdAb of the TriKE triggers degranulation even when expression of CD16 is lower than normal.

Limited cell numbers prevented us from enriching NK cells from patient PBMC in order to perform time-lapse imaging of cytotoxicity, as we had done for healthy donors (**Figure 5**). We were, however, able to use chromium release assays with whole PBMC to test whether the addition of TriKE to patient PBMC would lead to NCI-H460 cell lysis (**Figure 7C**). We tested this with the most at risk patient samples (stage IVB, at progression). The proportion of NK cells within the PBMC was between 8-25% for these samples, as determined by the CyTOF analysis. We increased the effector to target ratio of PBMC to tumor cells to account for this. Although thawed PBMC do not perform as well as freshly isolated cells in cytotoxicity assays, these patient samples were able to lyse NCI-H460 in the presence of TriKE, as well as or better than healthy donor cells, consistent with our degranulation results comparing healthy donors and lung cancer patient NK cells (**Figure 6**). Together these data suggest that TriKE has the capacity to induce robust responses against lung cancer cells even in patients that otherwise do not respond to treatment.

## Discussion

Chronic inflammation and associated lymphocyte exhaustion are important considerations in the treatment of cancer. In NSCLC, inflammation (as measured by CRP and neutrophil proportions), alongside serum lactate dehydrogenase and CA125, predict patient responses to chemotherapy (38), radiotherapy (39) and immunotherapy (40). The lymphocyte to monocyte ratio (41) is also predictive of patient outcomes in NSCLC – suggesting lymphocytes could be key to an effective response to treatment.

Given the importance of overcoming an exhausted or impaired immune system, mesothelin-targeted trials have pivoted in recent years to address this challenge. Most new mesothelin-targeting trials involve CAR T cells, with available results suggesting this is a safe, albeit costly, therapy (42–44). Another promising method of enhancing immune responses is cytokine therapy (45). Clinical trials have shown that IL-15 homologs safely expand patient NK cells and cytotoxic T cells (46–49). Importantly, IL-15 receptor agonists can be administered safely in combination with existing checkpoint immunotherapy to NSCLC patients (50) and clinical efficacy was seen in patients previously refractory to checkpoint blockade. Our TriKE molecule provides the benefit of cytokine therapy – enhanced proliferation and survival, with mesothelin-targeted cytotoxicity.

High avidity targeting of immune activating receptors in the context of bi-specifics is gaining traction as a therapeutic alternative to traditional antibody-based approaches to cancer immunotherapy (51). A CD3 and mesothelin targeting molecule (HPN536) has entered phase I clinical trials (NCT03872206); and preclinical models of a CD16 and mesothelin targeting bispecific molecule have demonstrated enhanced NK cell functions against triple negative breast cancer (52). Although the latter treatments lack the immune-stimulating effects of IL-15, they demonstrate that high avidity targeting of receptors can induce potent responses to a wide range of mesothelin-expressing cancers, not just NSCLC. Here, we have shown that low expression of CD16 on lung cancer patients undergoing treatment can be counteracted by high avidity CD16 molecules.

We described increased proportions of circulating myeloid cells, with a phenotype consistent with MDSC, after the onset of treatment in stage I-IVA NSCLC patients and at baseline in stage IVB NSCLC patients. Although MDSC are capable of suppressing NK cell cytotoxic function, we have previously shown that TriKE activation of NK cells is resistant to MDSC modes of suppression with the 161533 TriKE, which itself targets MDSC for cytotoxicity (53). Here we show that when this CD33+ CD14- myeloid population is present amongst PBMC, the cam1615SS1 TriKE consistently demonstrated enhanced NK cell activity in all patients and at all treatment stages.

We saw more CD56+ CD16+ NK cells in earlier stage NSCLC patients prior to treatment, who were less likely than stage IVB NSCLC patients to relapse. This observation is supported by another study that did not measure CD16 levels directly, but did find more CD56^dim^ NK cells amongst NSCLC patients that responded to PD-1 checkpoint immunotherapy (23). CD16 is found on CD56^dim^ cells in healthy donors, which suggests that we are observing common trends in NSCLC progression, despite a mixture of treatment regimens and disease definitions.

In this study TriKE was tested on NK cells from patient peripheral blood, not from the tumor. Lavin et al. (54) have shown that NK cells extracted from NSCLC tumors are a smaller proportion of the CD45+ population, have less CD16 and less granzyme B than NK cells from matched blood. We therefore do not know how tumor infiltrating NK cells would respond to TriKE. However, we have previously shown that NK cells derived from the ascites of ovarian cancer patients, which are less functional and express lower levels of CD16 than peripheral cells, respond to other TriKEs (55, 56). It is therefore feasible that, despite the decrease in CD16 expression, NSCLC intra-tumoral NK cells will still be responsive to TriKE. Furthermore, circulating NK cells (21–23) are predictive of a response to therapy, suggesting they are not irrelevant to therapy outcome. Circulating NK cells would have to migrate from the periphery in order to kill tumor cells. The IL-15 component in TriKEs enhances NK cell mobility *in vitro (* 53) and a TriKE targeting the tumor stroma was able to enhance NK cell infiltration into the tumor in an animal model (57). We would predict, therefore, that the IL-15 component of this TriKE, which targets lung cancer, would also enhance infiltration of NK cells directly into the tumor, but this remains to be shown. Once within the tumor microenvironment, many challenges remain to NK cell function, such as hypoxia (58), which were not investigated here.

There are potential challenges in the path towards anti-mesothelin TriKE becoming a viable treatment for NSCLC. Some anti-mesothelin and cytokine therapies have been hampered by patient-derived auto-antibodies. A clinical trial of a first generation CD33-targeted TriKE is underway (NCT03214666) and may reveal whether patients can develop antibodies against these biologics. The pre-clinical data provided here supports the proposition that mesothelin-targeted TriKE can drive proliferation of NK cells, killing of NSCLC cells and the release of inflammatory cytokines to co-ordinate robust immune responses. Together these data make mesothelin-targeted TriKE a promising candidate for NSCLC immunotherapy.

## Data availability statement

The raw data supporting the conclusions of this article will be made available by the authors, without undue reservation.

## Author contributions

PK, BE, CH, BK, DT, LB, PH, JW, BP acquired and analyzed the data. PK, DV, IP, RK, NF, JM, MF designed the work and interpreted the results. PK wrote the manuscript, DV, RK, NF, JM and MF revised the manuscript. All authors contributed to the article and approved the submitted version.
