# Supplementary material for: A tri-specific killer engager against mesothelin targets NK cells towards lung cancer
Source: Front Immunol. 2023 Feb 22;14:1060905. doi: 10.3389/fimmu.2023.1060905 (PMC9992642; doi:10.3389/fimmu.2023.1060905)
Supplement: Supplementary file 1 [file DataSheet_1.pdf]

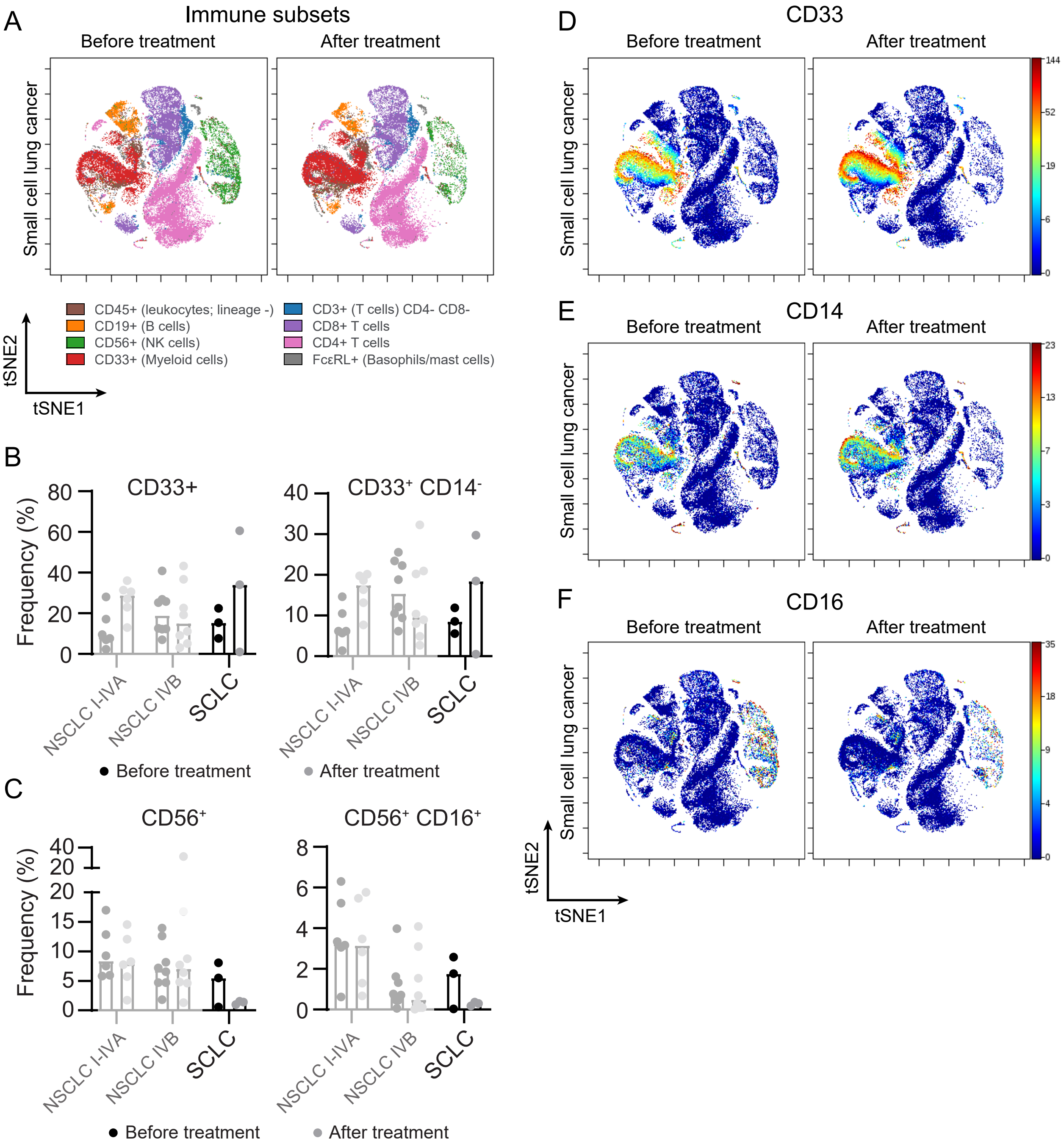


**Fig S1. Three small cell lung cancer patients have low circulating proportions of NK cells after treatment.** PBMCs from three small cell lung cancer patients were included along with the NSCLC PBMCs from the thoracic/lung nodule biobank. Analysis of these samples is included for reference alongside the NSCLC data. Matched samples were obtained for all patients before they began therapy and after initial treatment. (A) tSNE plots show concatenated patient samples before or after treatment onset, analyzed by CyTOF, for all CD45+ live cells, highlighting immune subsets as indicated by the cell surface markers. (B-C) The frequency of (B) CD33+ cells and CD33+ CD14- cells and (C) CD56+ and CD56+ CD16+, as a proportion of all CD45+ cells, is plotted for all patient groups (NSCLC patients are faded out) across both timepoints. (D-F) tSNEs shown in (A) are colored according to the intensity of (D) CD33, (E) CD14 or (F) CD16 staining.

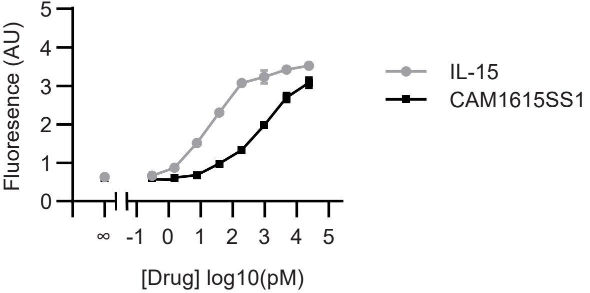


**Fig S2. Cam1615SS1 has a lower bioactivity than IL-15.** NK92 cells were deprived of IL-2, then stimulated with IL-15 or cam1615ss1 at a range of concentrations. After 40h, viable cells were quantified for their ability to convert resazurin into a fluorescent product. One representative experiment of three is shown. Mean values are plotted and bars represent the standard error of the mean (measured in quadruplicate). EC50 were calculated for IL-15 (22.0 pM) and TriKE (973 pM) using a non-linear four parameter variable slope least squares fit curve.

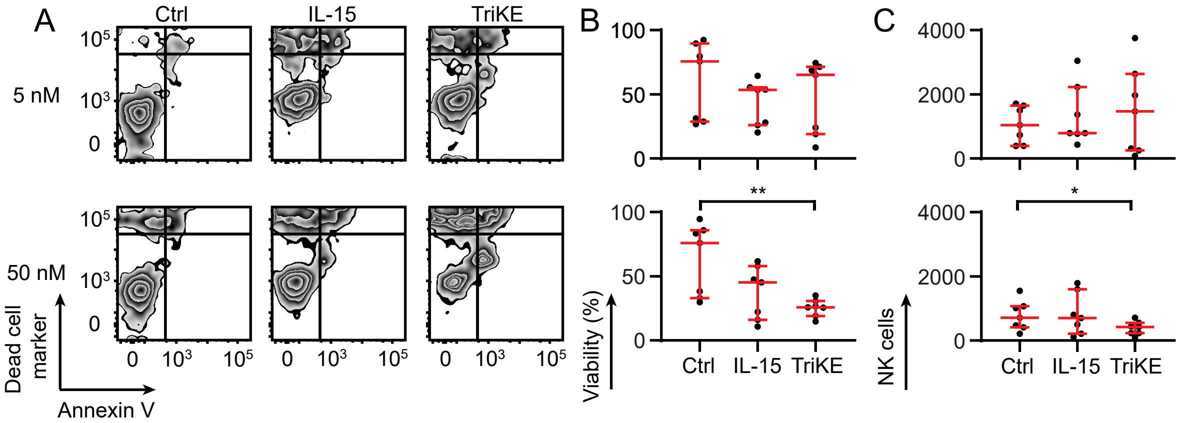


**Fig S3. At high doses, cam1615ss1 reduces viability of NK cells.** PBMCs were cultured in media without drugs (Ctrl), with IL-15 or with cam1615ss1 (TriKE) at the indicated concentrations. After 7 days, NK cells (CD56+ CD3-) were assessed by flow cytometry for viability (Annexin V$^-$ and dead cell marker$^-$). Representative zebra plots from one donor (A) are shown alongside quantification of viable cells for multiple donors (B). The number of NK cells at the end of the assay was also quantified (C) using a standardized volume, run at constant speed through the flow cytometer. Ctrl, IL-15 and TriKE (n=7) analysed by Friedman test with Dunn's multiple comparisons. Bars show median and interquartile range ** $p<0.01$, * $p<0.05$.

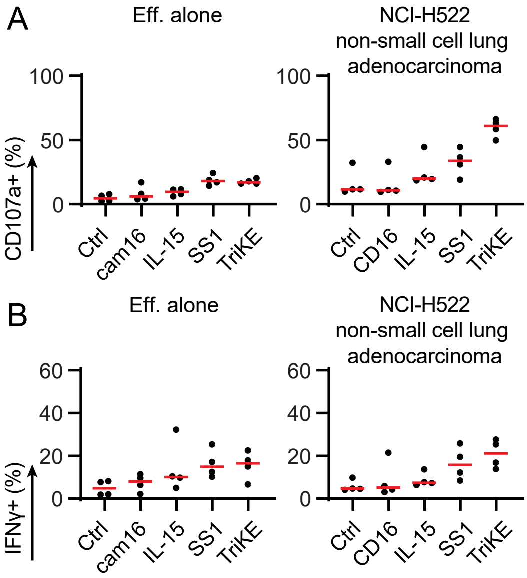


**Fig S4. Cam1615ss1 induces functional responses towards NCI-H522 lung cancer cells.** PBMCs were cultured in the presence of the indicated drugs (30nM), for 5 hours alone or in contact with or with NCI-H522. The amount of (A) degranulation (CD107a+) and (B) cytokine secretion (IFNγ) by NK cells (CD56+ CD3-) was assessed by flow cytometry. Each point represents a different donor (n=4). Bars show the median, no statistical test.

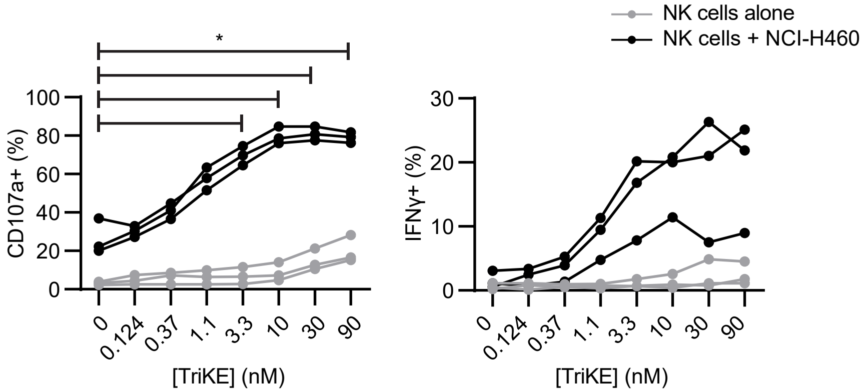


**Fig S5. 30nM of cam1615ss1 induces robust functional responses towards lung cancer cells.** PBMCs were cultured in the presence of cam1615ss1 (TriKE) at the indicated doses for 5 hours alone (grey, n=3) or with NCI-H460 (black, n=3). The amount of degranulation (CD107a+) and cytokine secretion (IFNγ) by NK cells (CD56+ CD3-) was assessed by flow cytometry. Each line represents a different donor. Two way repeated measures ANOVA with Dunnett's multiple comparisons (CD107a) or Sidak's multiple comparisons (IFNγ). * $p<0.05$.

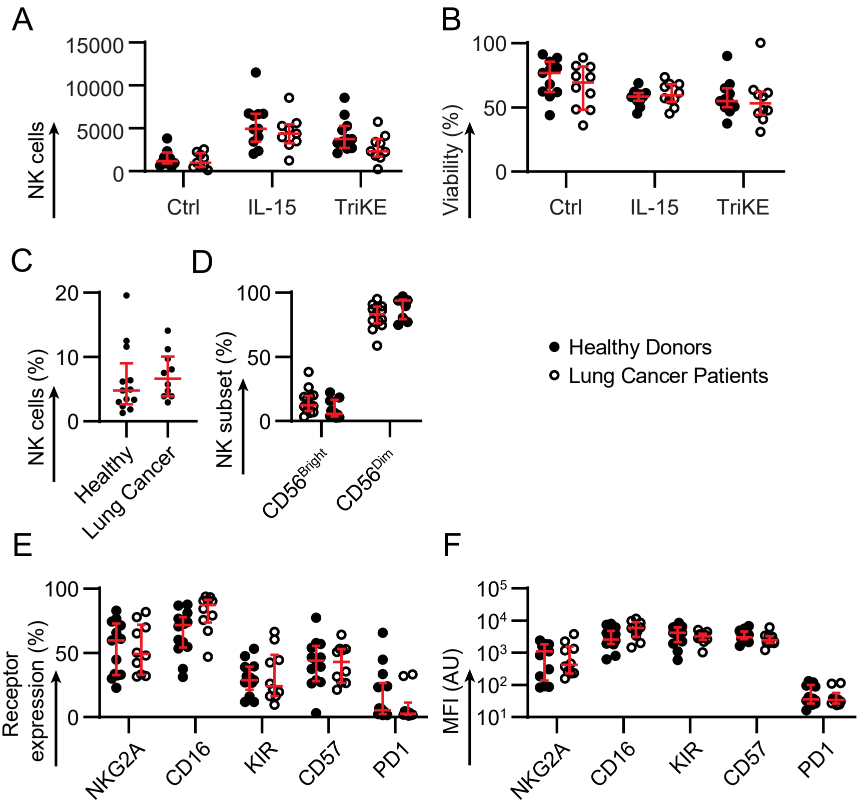


**Fig S6. Unstimulated NK cells have a similar phenotype and proliferative capacity in lung cancer patients and healthy donors.** Freshly isolated PBMCs from healthy donors (n=13) or lung cancer patients (n=10) were labelled with CellTrace Violet and cultured in media without drugs (Ctrl), with IL-15 or cam1615ss1 (TriKE) at 5nM (A-B). After 7 days (A) the number of NK cells was quantified using a standardized volume, run at constant speed through the flow cytometer and (B) their viability (Annexin V- and dead cell marker-) was also quantified. Proliferation data for this assay is shown in Figure 6A. (C-F) The freshly isolated samples were also stained for surface proteins. The frequency of (C) NK cells (CD56+ CD3-), (D) $CD56^{bright}$ and $CD56^{dim}$ subsets, or (E) lineage markers were assessed by flow cytometry. (F) The median fluorescence intensity (MFI) of receptors on NK cells expressing that receptor (KIR and CD57) or on all $CD56^{dim}$ NK cells (NKG2A, CD16, PD1) is also shown. A mixed effects analyses with Sidak's multiple comparison tests (A-B), Mann Whitney test (C), two-way ANOVA with Sidak's multiple comparison test (D) and mixed effects analyses with Sidak's multiple comparison tests (E-F) revealed no significant differences between healthy donors and lung cancer patients. Each dot represents a donor. Bars show median and interquartile range.
