# Supplementary material for: A tri-specific killer engager against mesothelin targets NK cells towards lung cancer
Source: Front Immunol. 2023 Feb 22;14:1060905. doi: 10.3389/fimmu.2023.1060905 (PMC9992642; doi:10.3389/fimmu.2023.1060905)
Supplement: Supplementary file 2 [file Table_1.docx]

Table S1: Patient characteristics (Figure 6 only)

|  | Cancer | NSCLC |
| --- | --- | --- |
|  | Patients | 10 |
|  | Median age (IQR) | 58 (56-69) |
| Sex | Female | 8 (80%) |
|  | Male | 2 (20%) |
| Ethnicity | White | 10 (100%) |
| Smoking* | Never | 1 (11%) |
|  | Former | 7 (78%) |
|  | Current | 1 (11%) |
| Staging | I-III | 6 (60%) |
|  | IVA | 1 (10%) |
|  | IVB | 3 (30%) |
| Histology (differentiation status) | Adenocarcinoma | 8 (4 poor, 1 moderate, 1 mucinous, 2 not specified) |
|  | Squamous | 2 (poor) |
|  | PDL1 % expression (range) | 0-90 |
| Treatments | Surgery | 5 (50%) |
|  | Chemotherapy | 4 (40%) |
|  | Radiation | 4 (40%) |
|  | Checkpoint inhibitor | 3 (30%) |
|  | Targeted therapy | 3 (30%) |
|  | Steroids | 0 (0%) |
| Complications | Immune-related adverse events | 3/3 (100%) |
| Progression | Progression (Y/N) | 4/6** (75%) |
|  | Died | 0/7 (0%) |
|  | Median time to 1st progression (months) | 9 (7-17) |
| Type of progression | Systemic | 3/4 (75%) |
|  | Central nervous system only | 2/4 (50%) |
| Best response to initial therapy | Complete response | 1/5 (20%) |
|  | Partial response | 5/5 (100%) |
|  | Stable disease | 1/5 (20%) |
|  | Progressive disease | 1/5 (20%) |
| Lost to follow up <1 year | | 3/10 (60%) |

Four patients overlap with patients in Table 1
* smoking information is missing for one patient
** four treated by surgery only, so progression data is not applicable

Table S2: CyTOF panel.

| Usage | Metal tag | Cat. | Company |
| --- | --- | --- | --- |
| Cisplatin | Pt195Di | 201064 | Fluidigm |
| Cell-ID™ Intercalator-Ir | Ir191Di,  Ir193Di | 201192A | Fluidigm |
| Cell-ID 20-Plex Pd Barcoding Kit | Pd102Di,  Pd104Di, Pd105Di, Pd106Di, Pd108Di,  Pd110Di | 201060 | Fluidigm |
| CD11B | Bi209Di | 3209003B | Fluidigm |
| CD127 | Nd143Di | 3143012B | Fluidigm |
| CD137 | Gd158Di | 3158013B | Fluidigm |
| CD14 | Eu151Di | 3151009B | Fluidigm |
| CD16 | Nd148Di | 3148004B | Fliudigm |
| CD19 | Nd142Di | 3142001B | Fluidigm |
| CD223 | Ho165Di | 3165037B | Fluidigm |
| CD226 | Yb171Di | 3171013B | Fluidigm |
| CD25 | Sm149Di | 3149010B | Fluidigm |
| CD27 | Gd155Di | 3155001B | Fluidigm |
| CD274 | Gd156Di | 3156026B | Fluidigm |
| CD3 | Er170Di | 3170001B | Fluidigm |
| CD314 | Er166Di | 3166016B | Fluidigm |
| CD33 | Dy163Di | 3163023B | Fluidigm |
| CD335 | Dy162Di | 3162021B | Fluidigm |
| CD337 | Tb159Di | 3159017B | Fluidigm |
| CD4 | Nd145Di | 3145001B | Fluidigm |
| CD45 | Y89Di | 3089003B | Fluidigm |
| CD56 | Yb176Di | 3163007B | Fluidigm |
| CD57 | Yb172Di | 3172009B | Fluidigm |
| CD62L | Eu153Di | 3153004B | Fluidigm |
| CD69 | Nd144Di | 3144018B | Fluidigm |
| CD8a | Nd146Di | 3146001B | Fluidigm |
| CD95 | Sm152Di | 3152017B | Fluidigm |
| FasLigand | Sm147Di | 201147A | Fluidigm |
| FcεRL |  | 334602 | Biolegend |
| (tagging kit) | Nd150Di | 201150A | Fluidigm |
| FoxP3 |  | 320102 | Biolegend |
| (tagging kit) | Dy161Di | 201161A | Fluidigm |
| Granzyme B | Yb173Di | 3173006B | Fluidigm |
| HLA-ABC |  | 311402 | Biolegend |
| (tagging kit) | Pr141Di | 201141A | Fluidigm |
| Ki-67 | Er168Di | 3168007B | Fluidigm |
| NKG2A | Tm169Di | 3169013B | Fluidigm |
| NKG2C | Gd160Di | 201160A | Fluidigm |
| NKp44 | Er167Di | 201167A | Fluidigm |
| PD-1 | Yb174Di | 3174020B | Fluidigm |
| Perforin | Lu175Di | 3175004B | Fluidigm |
| TIGIT | Sm154Di | 3209002B | Fluidigm |
| TRAIL | Dy164Di | 201164A | Fluidigm |
